# Circular RNA-VPS13A attenuates diabetes-induced enteric glia damage by targeting miR-182/GDNF axis: VPS13A attenuates glia damage by miR-182/GDNF

**DOI:** 10.3724/abbs.2022073

**Published:** 2022-07-08

**Authors:** Xiaowei Zhu, Yanyu Li, Xuping Zhu, Ke Wang, Xue Zhu, Yanmin Jiang, Lan Xu, Jianbo Li

**Affiliations:** 1 Department of Endocrinology and Metabolism the First Affiliated Hospital of Nanjing Medical University Nanjing 210029 China; 2 Department of Endocrinology Wuxi People′s Hospital Affiliated to Nanjing Medical University Wuxi 214000 China; 3 NHC Key Laboratory of Nuclear Medicine Jiangsu Key Laboratory of Molecular Nuclear Medicine Jiangsu Institute of Nuclear Medicine Wuxi 214063 China

**Keywords:** diabetes mellitus, gastrointestinal complications, enteric glia, circVPS13A

## Abstract

Gastrointestinal (GI) complications of diabetes mellitus (DM) significantly impact on patients’ quality of life. Enteric glial cells (EGC) are the key cell type of enteric nervous system (ENS), which contributes to the destruction of gut homeostasis in DM. Circular RNAs (circRNAs) are a novel type of RNAs abundant in the eukaryotic transcriptome, which form covalently closed continuous loops. In this study, the contribution of circRNAs to EGC damage in DM is investigated. Transcriptome sequencing analysis and functional study show that circVPS13A is significantly down-regulated in hyperglycemia-treated EGC, and circVPS13A overexpression attenuates EGC damage in both *in vitro* and *in vivo* DM models. *In vitro* mechanistic study using dual-luciferase reporter assay, affinity-isolation assay, fluorescence in situ hybridization (FISH) and immunostaining analysis identify that circVPS13A exerts its protective effect by sponging miR-182 and then up-regulates glial cell line-derived neurotrophic factor (GDNF) expression. In addition, *in vivo* study confirms that the circVPS13A-miR-182-GDNF network regulation can attenuate hyperglycemia-induced EGC damage of duodenum in streptozotocine (STZ)-induced DM mice. The findings of this study may provide novel insights into the protective role of circVPS13A in DM-associated EGC damage and clues for the development of new therapeutic approaches for the prevention of GI complications of DM.

## Introduction

Diabetes mellitus (DM) widely influences organs in the body, and gastrointestinal (GI) complications are more common in diabetic patients than in healthy subjects [ 1- 3] . Thus, better managing GI complications is important for the control of DM and the improvement of patients’ quality of life. The pathogenesis of GI complications in DM is complex. During its pathogenic process, dysfunction of enteric nervous system (ENS) is a leading cause with great clinical importance [ 4, 5] . Enteric glial cells (EGC) are the main type of cells in ENS, which form an extensive network in GI tract to protect enteric neurons [ 6, 7] . In a previous study, changes of the morphology and number of EGC were observed in the gastric motility dysfunction in DM [8]. In addition, it has been postulated that in the acute diabetes, EGC are activated to protect enteric neurons from damage via promoting neurotrophin release [9]. Therefore, enteric glia is responsible for gut motility reflexes, barrier function and inflammation in diabetes-associated GI complications. Understanding the mechanism underpinning enteric glia dysfunction in such pathogenetic process may contribute to exploring novel therapeutic approaches to better control GI complications associated with DM.

Circular RNAs (circRNAs) are a new class of noncoding RNAs, which are likely related to the occurrence and development of various human diseases [ 10, 11] . Thus, elucidating the functional role of circRNAs may contribute to the understanding of disease pathogenesis [ 12, 13] . Different types of non-coding RNAs including microRNAs (miRNAs) and long non-coding RNAs (lncRNAs) have been shown to be key regulators in the development of DM [ 14, 15] . However, little is known about the newly discovered circRNAs in the pathological process of DM. Lisa et al. [16] revealed that circRNAs are novel regulators of β-cell activities and suggested their involvement in β-cell dysfunction in DM. Caspase-1-associated circRNA (CACR) likely leads to diabetic cardiomyopathy (DCM) pyroptosis via the miR-214-3p/caspase-1 pathway [17]. Induction of cZNF532 regulates pericyte biology by acting as a miR-29a-3p sponge and inducing the expressions of NG2, LOXL2, and CDK2, which is an exploitable therapeutic approach for the treatment of diabetic retinopathy [18]. Up till now, it still remains unclear about the role of circRNAs in diabetes-associated GI complications.

In this study, we found that circVPS13A is significantly down-regulated in hyperglycemia-treated EGC based on transcriptome sequencing data, and further investigated the role of circVPS13A in the diabetes-induced enteric glia damage. This study may lead to a new therapeutic approach for the treatment of diabetes-associated GI complications.

## Materials and Methods

### Cell culture and transfection

CRL-2690 enteric glial cells were purchased from ATCC (Manassas, USA) and cultured in DMEM medium (Gibco, Carlsbad, USA) supplemented with 10% fetal bovine serum (Gibco) and 25 mM D-glucose (Sigma, St Louis, USA). Hyperglycemia concentration was obtained by adding D-glucose to a final concentration of 300 mM. For experiments, cells were transfected with GDNF siRNA1 (cat. no. sc-156116; Santa Curz, Dallas, USA), GDNF siRNA2 (cat. no. MBS8200788; Mybio, San Diego, USA), pcDNA3.1-circVPS13A (Genscript, Nanjing, China) using Lipofectamine 2000 (Life Technologies, Carlsbad, USA) according to the manufacturer’s protocols.

### TUNEL assay

Terminal deoxynucleotidyl transferase (TdT) dUTP Nick-End Labeling (TUNEL) assay Kit (Beyotime, Nantong, China) was used to detect apoptotic cells. Cells or tissues were fixed with 4% paraformaldehyde for 30 min and treated with 0.5% Triton X-100-PBS for 5 min according to the manufacturer′s instructions. Following this, the sections were then incubated with a terminal deoxynucleotidyl transferase reaction mix for 60 min at 37°C, followed by DAPI staining for 5 min. TUNEL‐positive cells (red staining cells) were observed via fluorescence microscopy (Olympus, Tokyo, Japan).

### STZ-induced diabetic mouse

C57BL/6J mice were purchased from Changzhou Cavens Experimental Animal Center (Changzhou, China). DM was induced by intraperitoneal injection of STZ (Sigma). Mice received STZ (50 mg/kg in 10 mM citrate buffer, pH 4.5) injection for 5 consecutive days. Control mice were administered with the same volume of sodium citrate buffer. All mice were kept in an air-conditioned room with a 12-hour light-dark cycle and fed with standard laboratory chow, with free access to water. Seven days after the final STZ injection, blood samples were collected from the tail vein for the measurement of blood glucose level. The mice with a blood glucose level of over 300 mg/dL were considered as DM. All animal experiments were performed in accordance with the institutional guidelines of Jiangsu Institute of Nuclear Medicine (Wuxi, China) for the Care and Use of Laboratory Animals, and this study was approved by the Ethics Committee of Jiangsu Institute of Nuclear Medicine.

### Transcriptome sequencing

CRL-2690 cells were incubated with 25 mM glucose or 300 mM glucose for 24 h. After incubation, RNA samples were prepared from cells for transcriptome sequencing by Novogene (Beijing, China) . The clustering of samples was processed through the cBot Cluster Generation System (Illumia, San Diego, USA) according to the manufacturer’s instructions. After cluster generation, the library was sequenced on the Illumina Hiseq platform and 125 bp/ 150 bp paired-end reads were generated. HTSeq v0.6.0 was used to count the number of reads mapped to each gene. FPKM of each gene was calculated based on the gene length with reads count mapped individually.

### Quantitative PCR analysis

Total RNA was isolated from cells using TRIzol reagent (TaKaRa, Dalian, China) and subjected to reverse transcription using a PrimeScript RT Master Mix Kit (TaKaRa). Quantitative PCR was performed using a Realtime PCR kit (TaKaRa) with the following primers: *circVPS13A* F: 5′-TGAAATTCTTGCAGAAATGTTG-3′, R: 5′-GTGCTCCTGGTCTTTGCACAAT-3′; *GDNF* F: 5′-TTCAAGCCACCATCAAAAGAC-3′, R: 5′-GTAGCCCAAACCCAAGTCAGT-3′; rat *GAPDH* F: 5′-TGCCACTCAGAAGACTGTGG-3′, R: 5′-TTCAGCTCTGGGATGACCTT-3′; mouse *GAPDH* F: 5′-TTGTCTCCTGCGACTTCAACAG-3′, R: 5′-GGTCTGGGATGGAAATTGTGAG-3′; *miR-182* F: 5′-ATCACTTTTGGCAATGGTAGAACT-3′, R: 5′-TATGGTTTTGACGACTGTGTGAT-3′; and *U6* F: 5′-GCTTCGGCAGCACATATACTAA-3′, R: 5′-AACGCTTCACGAATTTGCGT-3′. All reactions were run in triplicates. The level of miRNA was normalized to that of *U6*, and the relative levels of circular RNA were normalized to that of *GAPDH*. Total RNA from CRL-2690 cells was digested with RNase R (20 U, 37°C for 2 h) followed by qRT-PCR detection of circVPS13A and VPS13A mRNA expressions.

### Western blot analysis

Cells were treated with RIPA lysis buffer for 15 min prior to total protein extraction. Protein concentration was determined by BCA assay. Samples (20 μg) were subject to 10% sodium dodecyl sulfate polyacrylamide gel electrophoresis (SDS-PAGE) and transferred to polyvinylidene fluoride (PVDF) membrane (Millipore, Billerica, USA). The membrane was blocked with 5% bovine serum albumin (BSA) for 1 h and then incubated with the anti-GDNF antibody (1: 1000; Abcam, Cambridge, USA) or anti-GAPDH antibody (1:1000; Abcam), followed by incubation with the corresponding HRP-conjugated secondary antibody (1:500 ; Beyotime). The protein bands were visualized using an ECL assay kit (Beyotime) and quantified using ImageJ (National Institutes of Health, Bethesda, USA). GAPDH was used as the loading control.

### Enzyme‑linked immunosorbent assay (ELISA)

After treatment, the culture medium was collected and processed for ELISA. Culture medium (100 μL) was reacted with the GDNF ELISA kit (Solarbio, Beijing, China) according to the manufacturer′s instructions. The absorbance was measured at 450 nm using a microplate reader (Molecular Devices, San Jose, USA).

### Dual-luciferase reporter assay

TargetScan ( http://www.targetscan.org/) was used to predict the target of miR-182. Luciferase report assay was conducted for the measurement of miR-182 function. To evaluate the effect of miR-182 on circVPS13A, HEK293 cells were plated in 6-well plates and co-transfected with 100 pM of either miR-182 mimic (5′-UUUGGCAAUGGUAGAACUCACACCG-3′ or negative control (5′-UUCUCCGAACGUGUCACGUTT-3′) (Genepharma, Shanghai, China), 40 ng of either pGL3-circVPS13A or pGL3-circVPS13A-MUT ( Genepharma), and 4 ng of pRL-TK (Promega, Madison, USA) using Lipofectamine 2000 (Life Technologies). To assess the effect of miR-182 on GDNF, HEK293 cells were co-transfected with 100 pM of either miR-182 mimic or control, 40 ng of either pGL3-GDNF-3′UTR or pGL3-GDNF-3′UTR MUT (Genepharma), and 4 ng of pRL-TK (Promega) using Lipofectamine 2000 (Life Technologies). Forty-eight hours after transfection, cells were collected and analyzed using the Dual-luciferase Reporter Assay System (Promega).

### RNA pull-down assay

CRL-2690 cells were transfected with 3′-end biotinylated miR-182 (Genepharma) at a final concentration of 50 nM for 24 h. Then, the cells were lysed and centrifuged as described previously [19]. A total of 50 μL aliquots of the samples were prepared as input. The remaining lysates were incubated with M-280 streptavidin magnetic beads (Invitrogen, Carlsbad, USA) for 2.5 h at 4°C. Then the beads were washed twice with ice-cold lysis buffer, twice with low-salt buffer, and once with high-salt buffer. The bound RNAs were isolated using TRIzol reagent for the measurement of circVPS13A by qPCR.

### Fluorescence *in situ* hybridization (FISH) analysis

RNA FISH experiments were carried out using the kit purchased from Genepharma according to the manufacturer′s instructions. The probes of circVPS13A and miR-182 (labeled with Cy3 dye) were as follows: circVPS13A, CAAGAATACCATGTATAGCATAAGCCCACTTTAGGGCTTGTGCTCCTGGTCTTTGCAC; miR-182, CGGTGTGAGTTCTACCATTGCCAAA. All hybridizations were done overnight in the dark at 37°C in a humidified chamber. For protein FISH experiments in cells, CRL-2690 cells were cultured on coverslips, fixed with 4% paraformaldehyde for 20 min and incubated in PBS overnight at 4°C, followed by the detection of circVPS13A and miR-182 as previously reported [19]. The coverslips were heated to 65°C for 5 min in hybridization buffer containing digoxigenin-labeled miR-182 or biotin-labeled circVPS13A probe, and then hybridized overnight at 37°C. The coverslips were washed and blocked for 1 h at room temperature. After that, coverslips were incubated with a Cy3-conjugated anti-digoxigenin antibody overnight at 4°C and then cultured with FITC-streptavidin for 2 h at 4°C. The cell nuclei were stained with DAPI. Finally, slides were examined under a fluorescence microscope (Olympus) and digital images were captured.

### Digital PCR analysis

The chip-based digital PCR (dPCR) was conducted on a QuantStudio 3D Digital PCR System (Life Technologies) for the absolute quantification of the copy number of circVPS13A and miR-182. cDNA was synthesized from RNA isolated from EGC under indicated conditions, and digital PCR was conducted using the following reaction conditions: hot start at 96°C for 10 min, denaturation at 98°C for 30 s, annealing/extension at 62°C for 2 min for a total of 39 cycles, followed by a final extension step at 65°C for 2 min. The data analysis was performed with QuantStudio 3D AnalysisSuite Cloud Software version 3.1.2. The primers used are: *circVPS13A* F, 5′-TGAAATTCTTGCAGAAATGTTG-3′, R, 5′-GTGCTCCTGGTCTTTGCACAAT-3′; *miR-182* F, 5′-ATCACTTTTGGCAATGGTAGAACT-3′, R, 5′-TATGGTTTTGACGACTGTGTGAT-3′.

### Immunofluorescence analysis

The section of mouse duodenum was cut into 35-μm slices with a cryostat. Then, the sections were incubated with anti-GFAP antibody (Sigma) overnight at 4°C. On the following day, the sections were washed and incubated with Alexa Fluor 488-conjugated anti-mouse IgG for 1 h. After wash with PBS, the sections were incubated with ProLong gold Antifade reagent containing DAPI (Invitrogen) for visualization of the nuclei. Immunofluorescence images were captured with an Olympus DP73 fluorescence microscope (Olympus).

### Intestinal intramuscular injection

Virus injections were performed at 2 weeks after the establishment of DM mice model. The mice were divided into three groups: DM group, DM+circVPS13A OV (Overexpression), DM+circVPS13A OV+miR-182 TF (Transfection). Mice were anesthetized using isoflurane (2%–4%) and kept at a constant body temperature. For the experiments, circVPS13A was subcloned into pHBAAV-CMV- circRNA-EF1-ZsGreen vector, and the vector or micrON™ miR-182 agomir was packaged by AAV9, then the injection of 2 μL AAV9-circVPS13A (1.2×10 ^11^ GC/ml) or AAV9-miR-182 agomir (1×10 ^11^ GC/ml) was conducted using a 10-μL Hamilton syringe into the wall of the duodenum at two sites near the myenteric plexus as previously reported [20]. Two weeks after intestinal intramuscular injection, the efficacy of AAV9 delivery was assessed by evaluating the expressions of circVPS13A or miR-182 in duodenum by qPCR and RNA FISH.

### Statistical analysis

SPSS19.0 software was used to analyze data. Data were expressed as the mean±SD. Statistical comparisons were made by Student’s *t*-test between two groups and one-way ANOVA followed by Tukey’s post hoc test among different groups. *P*<0.05 indicates statistically significant difference.

## Results

### circVPS13A expression is significantly down-regulated in both *in vitro* and *in vivo* DM models

**Figure 1:**
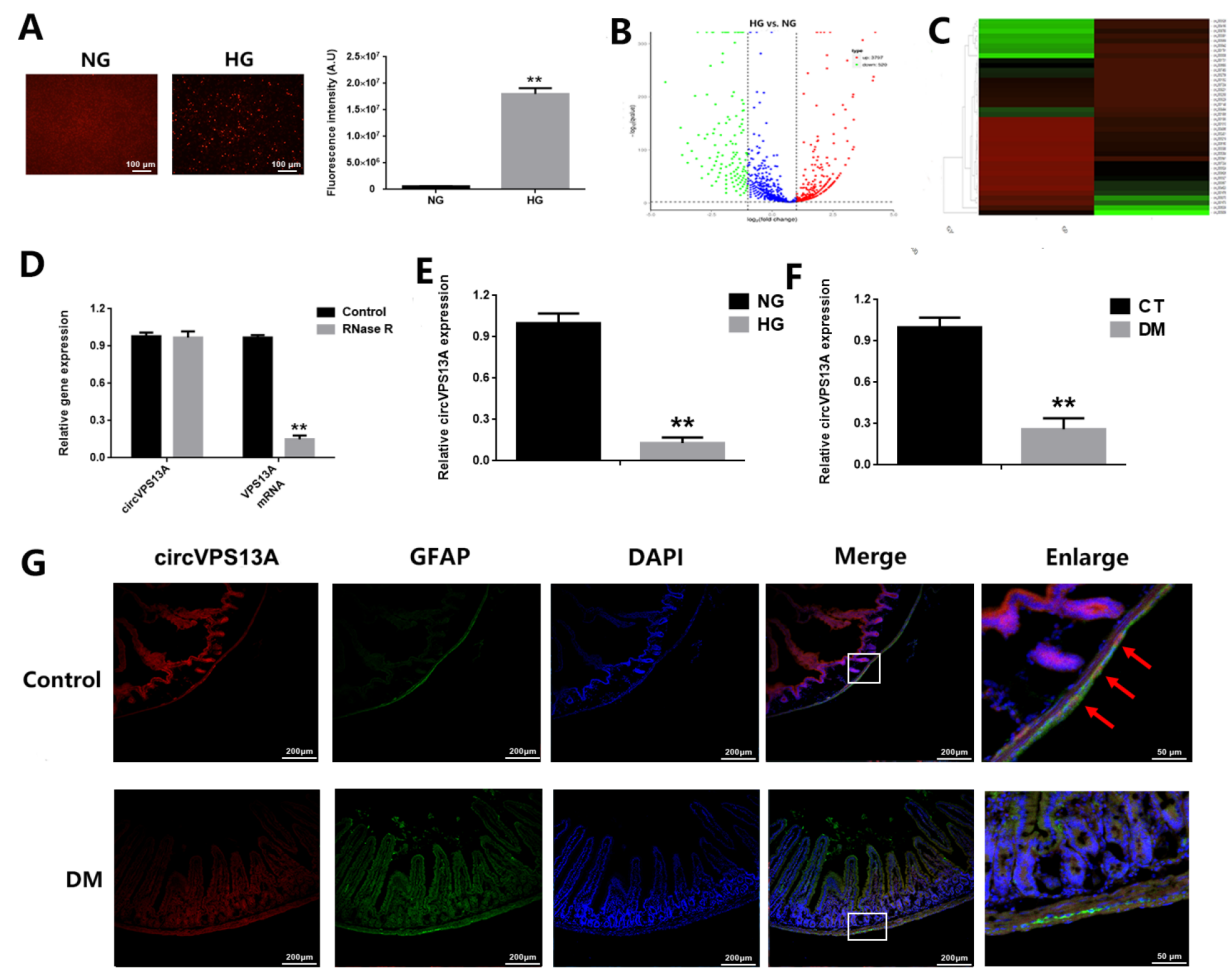
Figure 1 The expression of circVPS13A in *in vitro* and *in vivo* DM models (A) CRL-2690 cells were treated with 25 mM glucose (normal glucose, NG) or 300 mM glucose (high glucose, HG) for 24 h, and cell apoptosis was assessed by TUNNEL assay, and quantitatively analyzed. (B,C) CRL-2690 cells were treated with 25 mM glucose (normal glucose, NG) or 300 mM glucose (high glucose, HG) for 24 h, and transcriptome sequencing of circRNAs was conducted. Volcano plot and EGC Heatmap of significantly-changed circRNAs were presented. (D) CRL-2690 cells were treated with or without RNase R (20 U, 37°C for 2 h), and the relative expressions of circVPS13A and VPS13A mRNA were analyzed by qPCR. (E) CRL-2690 cells were treated with 25 mM glucose (normal glucose, NG) or 300 mM glucose (high glucose, HG) for 24 h, and the relative expression of circVPS13A was analyzed by qPCR. (F) The relative expression of circVPS13A in duodenum of control and DM mice (6 weeks post STZ-injection) was analyzed by qPCR. (G) The expression of circVPS13A in EGC of control and DM mice (6 weeks post STZ-injection) was analyzed by RNA FISH analysis. ** P<0.01.

To identify hyperglycemia-regulated circRNAs, CRL-2690 cells were cultured with 25 mM glucose (normal glucose, NG) or 300 mM glucose (high glucose, HG) for 24 h. First, cell viability was assessed by TUNEL assay and the results showed that HG significantly reduced cell viability in CRL-2690 cells ( Figure 1A). The RNA samples of NG and HG groups were prepared for transcriptome sequencing, and 4317 differentially expressed circRNAs were identified between the HG group and NG group, with 3797 upregulated and 520 downregulated ( Figure 1B). Among these altered circRNAs, circ_0003641 was found to be the most down-regulated circRNA, which has a homologous gene named VPS13A in rat genome with a sequence similarity of over 85% ( Figure 1C). circVPS13A was found to be resistant to RNase R digestion, whereas linear VPS13A mRNA responded to RNase R readily ( Figure 1D). Whether hyperglycemia influences circVPS13A expression *in vitro* and *in vivo* was further evaluated. qRT-PCR revealed that HG reduced circVPS13A expression in CRL-2690 cells after 24 h of treatment ( Figure 1E). Meanwhile, reduced circVPS13A was observed in the duodenum and EGC of diabetic mice at 6 weeks post STZ-induction ( Figure 1F,G). These data indicated that hyperglycemia significantly down-regulated circVPS13A expression in both *in vitro* and *in vivo* DM models.

### circVPS13A overexpression attenuates EGC damagein both *in vitro* and *in vivo* DM models

**Figure 2:**
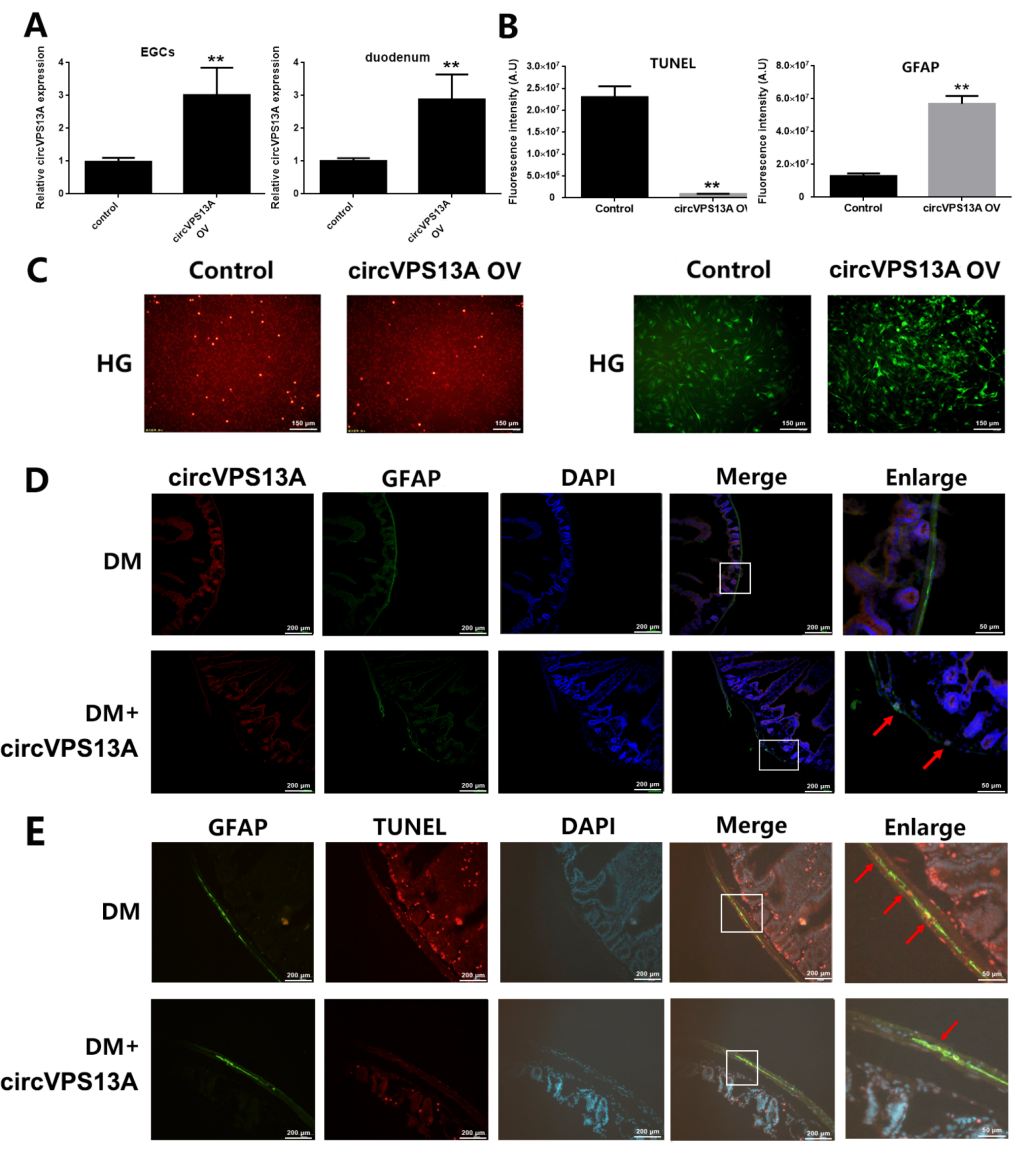
Figure 2 The inhibitory effect of circVPS13A overexpression on EGC damage in *in vitro* and *in vivo* DM models (A) CRL-2690 cells were transfected with pcDNA3.1-circVPS13A using lipofectamine 2000 and overexpression of circVPS13A was assessed by qPCR. DM mice (6 weeks post STZ-injection) were injected with AAV9-circVPS13A, and overexpression of circVPS13A in duodenum was assessed by qPCR after 2 weeks. (B) CRL-2690 cells were transfected with pcDNA3.1-circVPS13A using lipofectamine 2000, cell apoptosis was assessed by TUNNEL assay and the expression of GFAP was assessed by immunofluorescence assay and quantitatively analyzed. (C) Representative images of TUNNEL assay and GFAP immunofluorescence assay in CRL-2690 cells. (D) The co-expression of circVPS13A and GFAP was assessed by RNA FISH and immunofluorescence assay in duodenum of DM mice (6 weeks post STZ-injection). (E) The effect of circVPS13A overexpression on EGC damage in DM mice (6 weeks post STZ-injection). HG: high glucose, DM: diabetes mellitus. ** P<0.01.

To understand the function of circVPS13A under hyperglycemia, circVPS13A was up-regulated by circVPS13A overexpression in both *in vitro* and *in vivo* DM models ( Figure 2A). First, circVPS13A overexpression reduced the cytotoxicity and apoptosis of CRL-2690 cells under hyperglycemia ( Figure 2B,C). In addition, the up-regulated expression of glia marker GFAP was observed, indicating that circVPS13A attenuated EGC damage under hyperglycemia ( Figure 2B,C). Then, the inhibitory effect of circVPS13A overexpression on EGC damage in STZ-induced diabetic mice was evaluated by intestinal intramuscular injection of AAV packaged circVPS13A expression plasmid. The injection up-regulated the expression of circVPS13A in EGC of duodenum, and subsequently reduced apoptosis of EGC and increased EGC density *in vivo* ( Figure 2D,E). These data indicated that hyperglycemia-induced EGC damage could be attenuated by circVPS13A overexpression in both *in vitro* and *in vivo* DM models.

### circVPS13A attenuates EGC damage by acting asa sponge for miR-182

**Figure 3:**
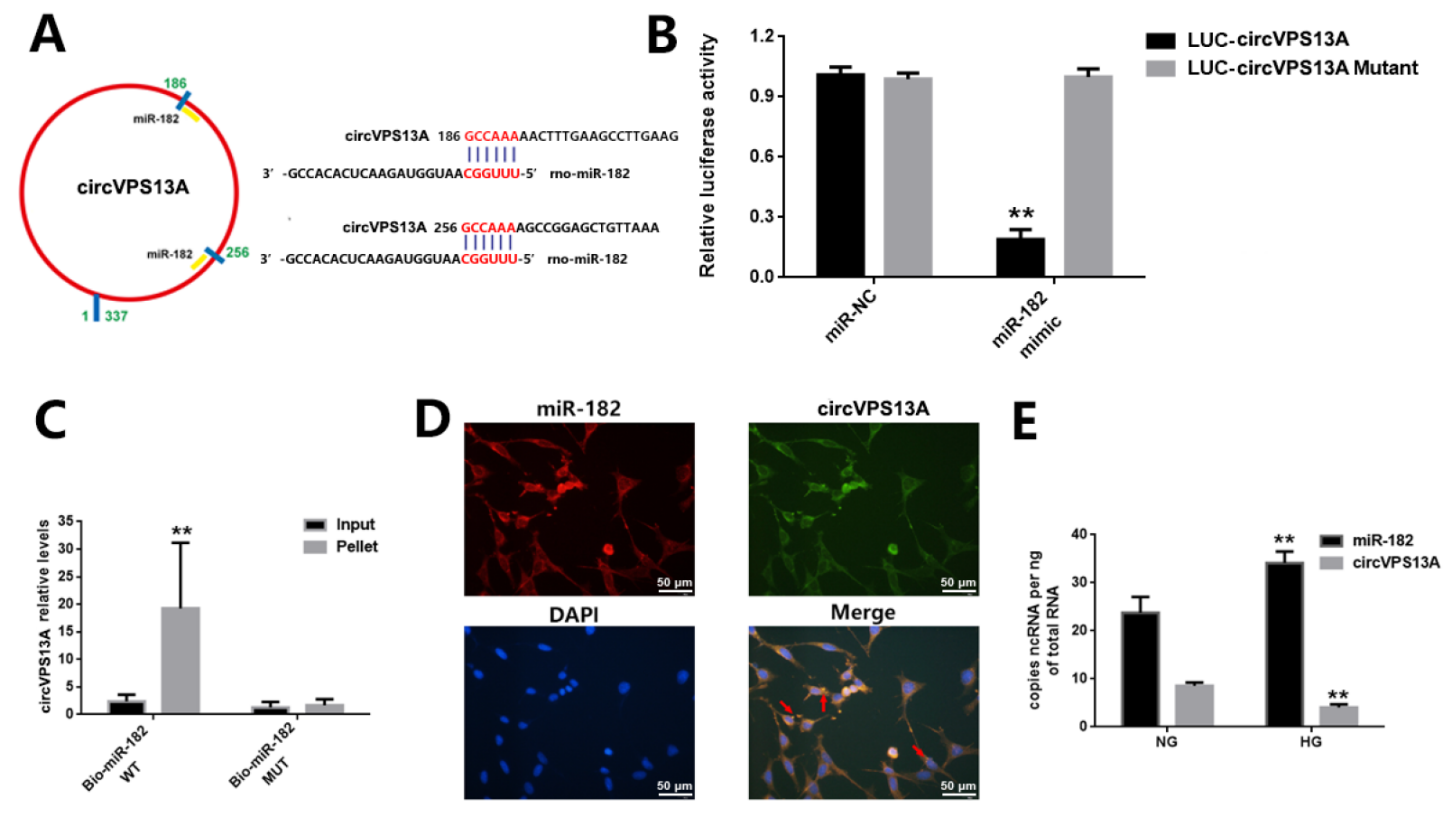
Figure 3 The regulatory effect of circVPS13A on EGC damage by acting as a miR-182 sponge (A) The predicted binding site for miR-182 in circVPS13A transcript. (B) The luciferase reporter vector containing circVPS13A or circVPS13A mutant and miR-NC or miR-182 mimic were co-transfected into HK293T cells, and luciferase activity was determined and normalized to Renilla luciferase activity. (C) RNA pull-down assay was conducted using biotinylated miR-182 in CRL-2690 cells. (D) FISH assay was conducted using in situ hybridization of circVPS13A and miR-182 in CRL-2690 cells. (E) Digital PCR was conducted for the absolute quantification of circVPS13A and miR-182. ** P<0.01.

**Figure 4:**
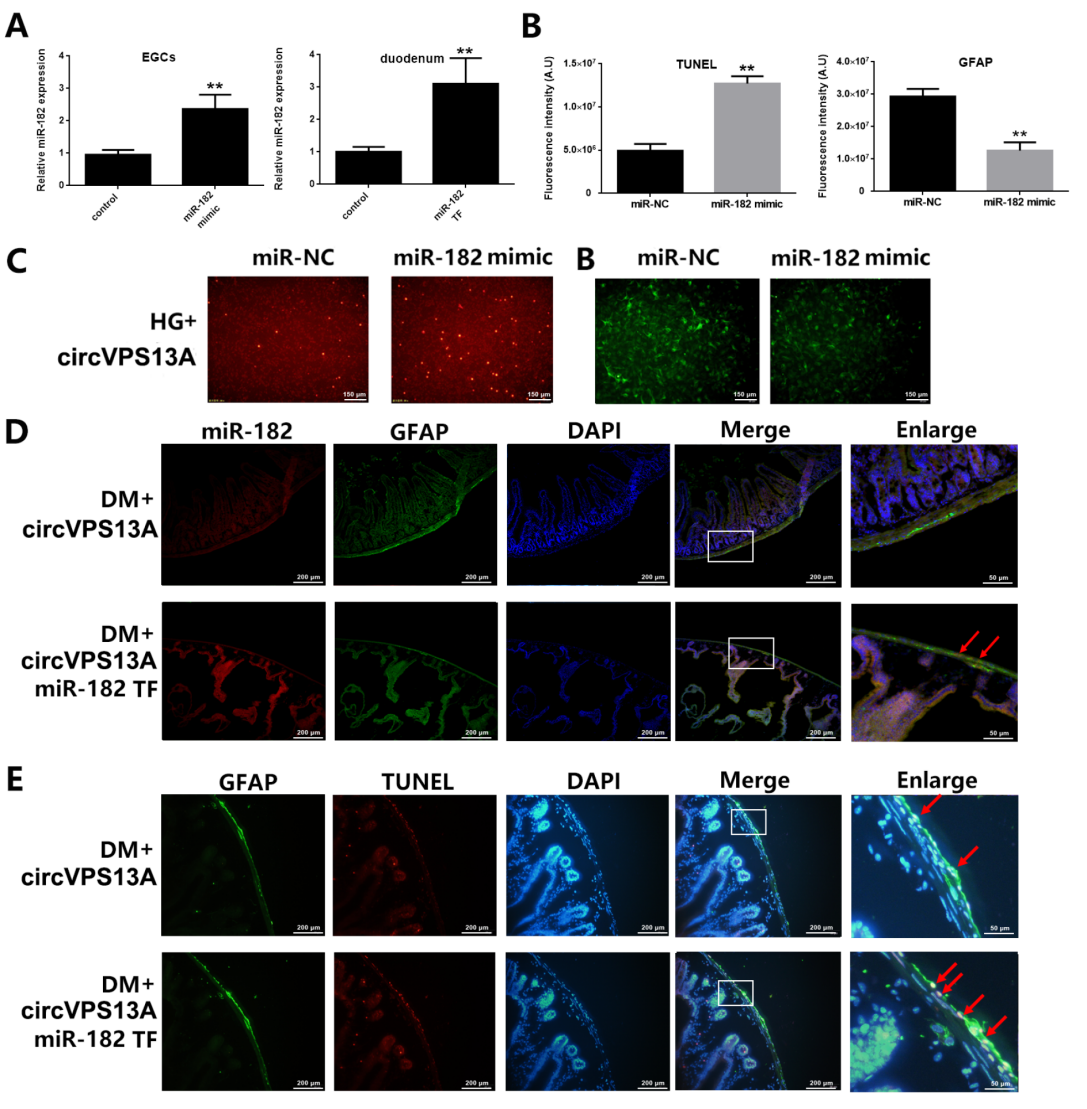
Figure 4 The reversal effect of miR-182 overexpression on the molecular consequences of circVPS13A overexpression in *in vitro* and *in vivo* DM models (A) CRL-2690 cells were transfected with miR-182 mimic using lipofectamine 2000 and overexpression of miR-182 was assessed by qPCR. DM mice (6 weeks post STZ-injection) were injected with AAV9-miR-182 agomir, and overexpression of miR-182 in duodenum was assessed by qPCR after 2 weeks. (B) CRL-2690 cells were transfected with miR-182 mimic using lipofectamine 2000, cell apoptosis was assessed by TUNNEL assay and the expression of GFAP was assessed by immunofluorescence assay and quantitatively analyzed. (C) Representative images of TUNNEL assay and GFAP immunofluorescence assay in CRL-2690 cells. (D) The co-expression of miR-182 and GFAP was assessed by RNA FISH and immunofluorescence assay respectively in duodenum of DM mice (6 weeks post STZ-injection). (E) The effect of miR-182 overexpression on EGC damage in DM mice (6 weeks post STZ-injection) with circVPS13A overexpression. HG: high glucose; DM: diabetes mellitus; TF: transfection.** P<0.01.

To understand the mechanism of the regulatory role of circVPS13A under hyperglycemia, the target genes of circVPS13A were analyzed using bioinformatics tools and dual-luciferase reporter assay. First, bioinformatics data predicted that circVPS13A exerts its function at the post-transcriptional level by acting as a miR-182 sponge ( Figure 3A). Then, dual-luciferase reporter assay identified that transfection with miR-182 mimic decreased the luciferase activity of LUC-circVPS13A without influencing the luciferase activity of LUC-circVPS13A mutant ( Figure 3B). Finally, RNA pull-down assay showed that circVPS13A was enriched in the miR-182-captured fraction ( Figure 3C). FISH assay revealed the co-expression of circVPS13A and miR-182 in the cytoplasm of CRL-2690 cells ( Figure 3D), and digital PCR showed the copies of circVPS13A were significantly down-regulated and the copies of miR-182 were slightly up-regulated in hyperglycemia-treated EGC compared to controls ( Figure 3E). In addition, miR-182 overexpression reversed the protective effect of circVPS13A overexpression on hyperglycemia-induced EGC damage ( Figure 4). These data indicated that circVPS13A attenuated hyperglycemia-induced EGC damage by acting as a sponge for miR-182

### circVPS13A-miR-182-GDNF network is involved in regulating EGC damage in both *in vitro* and *in vivo*DM models

**Figure 5:**
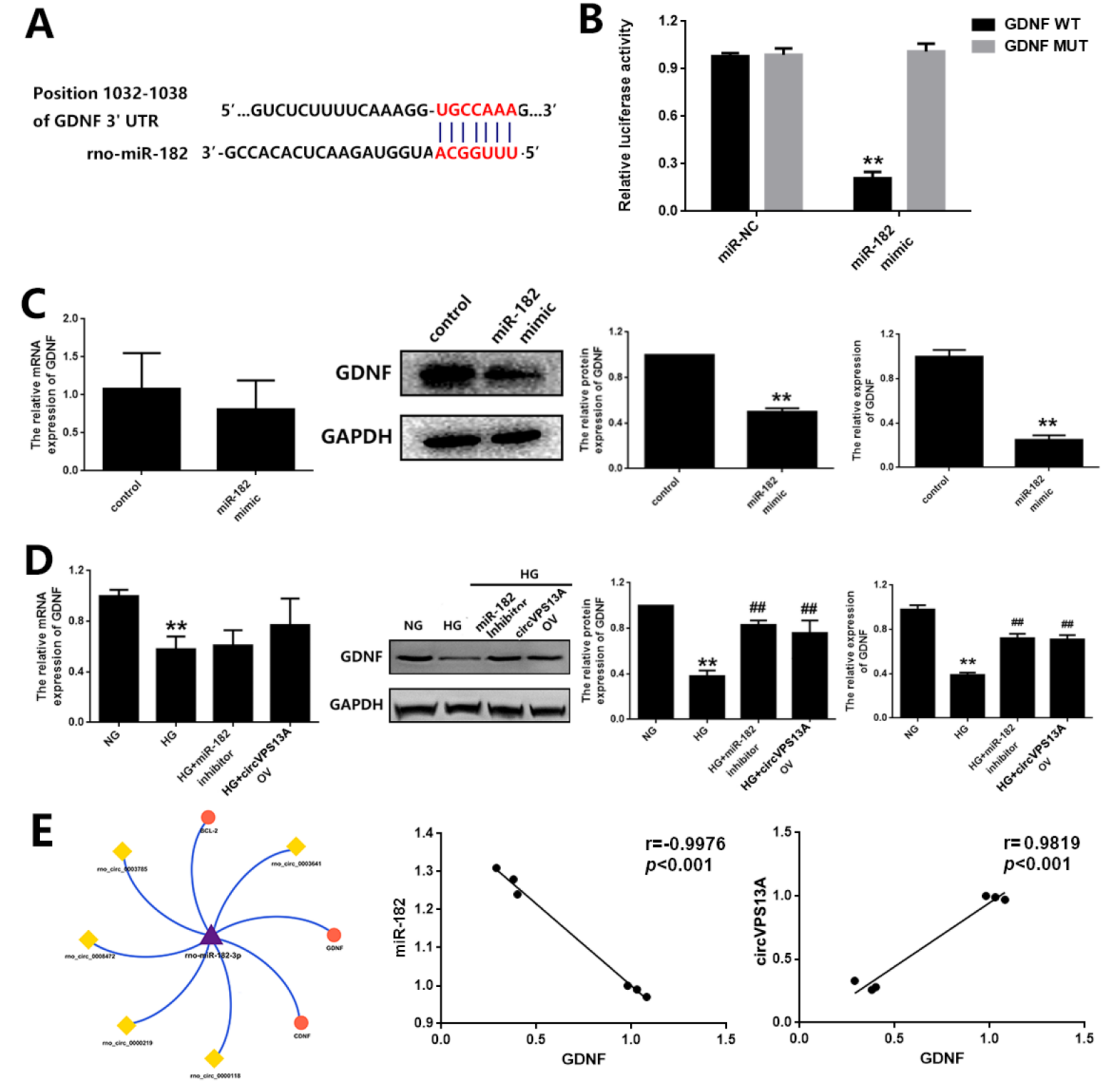
Figure 5 The role of GDNF in the regulatory effect on circVPS13A-miR-182 on EGC damage in DM (A) The predicted binding site for miR-182 in the 3’-UTR of GDNF. (B) The luciferase reporter vector containing WT GDNF 3’-UTR or MUT GDNF 3’-UTR and miR-NC or miR-182 mimic were co-transfected into HK293T cells, and luciferase activity was determined and normalized to Renilla luciferase activity. (C) The effect of transfection with miR-182 mimic on GDNF expression in CRL-2690 cells was assessed by qPCR, western blot analysis, and ELISA. (D) The effect of miR-182 inhibitor or circVPS13A overexpression on GDNF expression in HG-treated CRL-2690 cells by qPCR, western blot analysis, and ELISA. (E) The correlation analysis of GDNF with miR-182 or circVPS13A in CRL-2690 cells with or without HG treatment. ** P<0.01; ## P<0.01.

**Figure 6:**
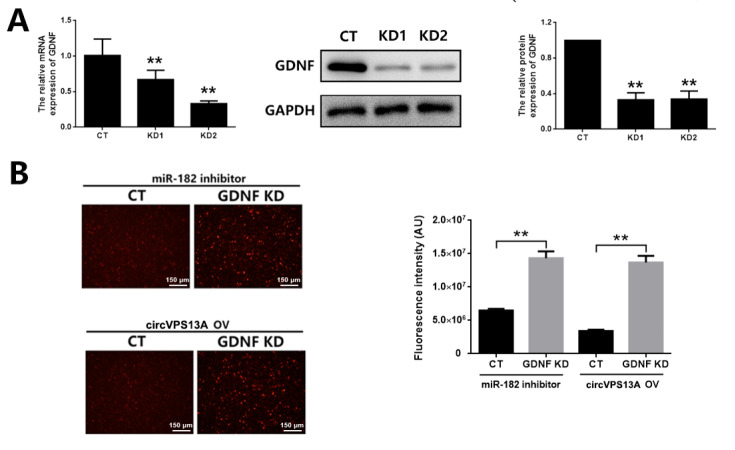
Figure 6 The effect of miR-182 inhibitor or circVPS13A overexpression on cell apoptosis in HG-treated CRL-2690 cells with or without *GDNF* knockdown (A) CRL-2690 cells were transfected with GDNF siRNA using lipofectamine 2000, and GDNF knockdown was assessed by qPCR, western blot analysis, and ELISA. (B) CRL-2690 cells were transfected with GDNF siRNA using lipofectamine 2000, and cell apoptosis was assessed by TUNEL assay and quantitively analyzed. NG: normal glucose; HG: high glucose; CT: control; KD: knockdown. ** P<0.01.

**Figure 7:**
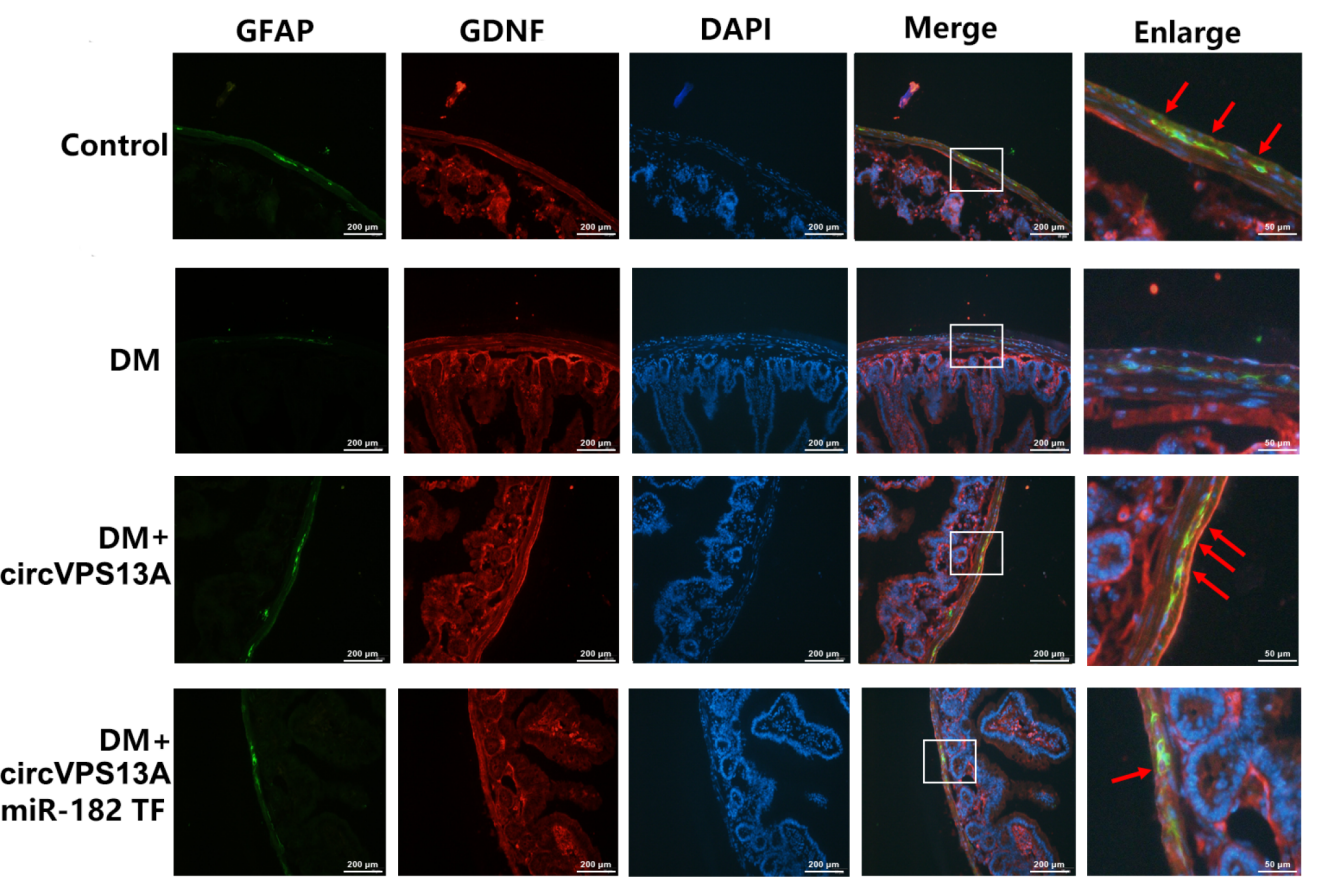
Figure 7 The effect of circVPS13A-miR-182 on GDNF expression in EGC in *in vivo* DM models STZ-mice (6 weeks post STZ--injection) were divided into four groups: Control, DM, DM with circVPS13A overexpression, and DM with circVPS13A+miR-182 overexpression. Then, the expression of GDNF in EGC of duodenum in DM mice was assessed by immunofluorescence assay.

Targetscan database was used to predict the target genes of miR-182. *GDNF* is one of the candidate genes to be selected for further evaluation due to its role in glia activation. Luciferase reporter assay verified the direct regulation of miR-182 in GDNF expression; while miR-182 mimic significantly reduced GDNF expression at the protein level ( Figure 5A–C). In addition, GDNF expression was down-regulated in hyperglycemia-treated EGC, and negatively correlated with miR-182 but positively correlated with circVPS13A ( Figure 5D,E). Further study also revealed that miR-182 inhibitor or circVPS13A overexpression significantly enhanced GDNF expression at the protein level in hyperglycemia-treated EGC ( Figure 5D). Meanwhile, *GDNF* silencing attenuated the protective effect of miR-182 inhibitor or circVPS13A overexpression on hyperglycemia-induced EGC cytotoxicity and apoptosis in CRL-2690 cells ( Figure 6), indicating that circVPS13A overexpression acts similarly to that of miR-182 inhibitor. Finally, the role of the circVPS13A-miR-182-GDNF network in EGC damage was investigated in diabetic mice. circVPS13A overexpression led to the increased expression of GDNF, which was attenuated by transfection with miR-182 agomir ( Figure 7). These data indicated that the circVPS13A-miR-182-GDNF network regulation could attenuate hyperglycemia-induced EGC damage in both *in vitro* and *in vivo* DM models.

## Discussion

Impaired function of the GI tract is the common complication of DM, and EGC dysfunction contributes to the destruction of gut homeostasis under pathophysiological conditions [ 21, 22] . Therefore, it is urgent to develop new therapeutic strategies to control diabetes-related GI complications. In this study, we demonstrated that circVPS13A was significantly down-regulated in CRL-2690 cells under hyperglycemia based on transcriptome sequencing and qPCR, and that circVPS13A overexpression attenuated EGC damage in both *in vitro* and *in vivo* DM models. Furthermore, circVPS13A acted as a miR-182 sponge to sequester and inhibit miR-182 activity, which led to increased expression of GDNF. This study might provide novel insights into the understanding of the pathogenesis of DM-related GI complications.

circRNAs exert their functions by acting as the sponges for miRNAs or proteins, competing with the linear mRNA, or regulating the transcription of their parental genes [ 23, 24] . circRNAs are critically involved in metabolic processes of gestational diabetes mellitus (GDM) and related to insulin resistance and β-cell dysfunction [ 25, 26] . VPS13A was previously identified as a key factor in various human neurodegenerative diseases [ 27, 28] . And up to now, no literature showed the crypt expression of VPS13A in the small intestine and colon. Therefore, little is known about the function of circular RNA-VPS13A in the gut. In this study, we revealed that increased circVPS13A could sponge and sequester miR-182 in response to hyperglycemia, relieving its miR-182-mediated suppressive effect on EGC damage. MiR-182 plays a significant role in regulating neuronal axon outgrowth and dendrite tree formation [29]. Roser *et al*. [30] demonstrated that inhibition of miR-182 in GDNF-treated polymorphonuclear neutrophils (PMN) cultures diminished the beneficial effect of GDNF, suggesting that miR-182 is involved in mediating the effects of GDNF. Here, we found that hyperglycemia enhanced the expression of miR-182, and that inhibition of miR-182 attenuated hyperglycemia-induced EGC damage both *in vitro* and *in vivo*, which is comparable to that of circVPS13A overexpression.

To further investigate the effects of miR-182, targetscan database and luciferase reporter assay were performed to confirm whether GDNF, an important factor in glia activation, is the direct target of miR-182. GDNF is known to participate in the regulation of EGC function, and altered GDNF expression leads to a higher susceptibility towards apoptosis, resulting in the disruption of the mucosal integrity and severity of inflammation [31]. Zhang *et al*. [32] reported that EGC can regulate intestinal epithelial barrier integrity indirectly via manipulating the release of GDNF *in vivo* [32]. Bauman *et al*. [33] showed that EGC lost their barrier-enhancing function under morphine treatment due to the decreased production of GDNF [33]. In the current study, we also found that GDNF was significantly down-regulated in hyperglycemia-treated EGC; while circVPS13A overexpression or miR-182 inhibition significantly enhanced GDNF expression. Consistently, *GDNF* silencing attenuated the protective effect of circVPS13A overexpression or miR-182 inhibition on hyperglycemia-induced EGC cytotoxicity and apoptosis in CRL-2690 cells. In addition, our *in vivo* study confirmed the protective effect of circVPS13A overexpression on EGC damage. However, future studies are required on the *GNDF* knockdown mouse model to further investigate the molecular mechanism of circVPS13A/miR-182. In addition, besides modulating circVPS13A-miR-182 signaling to enhance GDNF in EGC, normalizing GFAP+ enteric glial cells could also recover GDNF expression indirectly in duodenum, since enteric glial cells are a source of GDNF.

**Figure 8:**
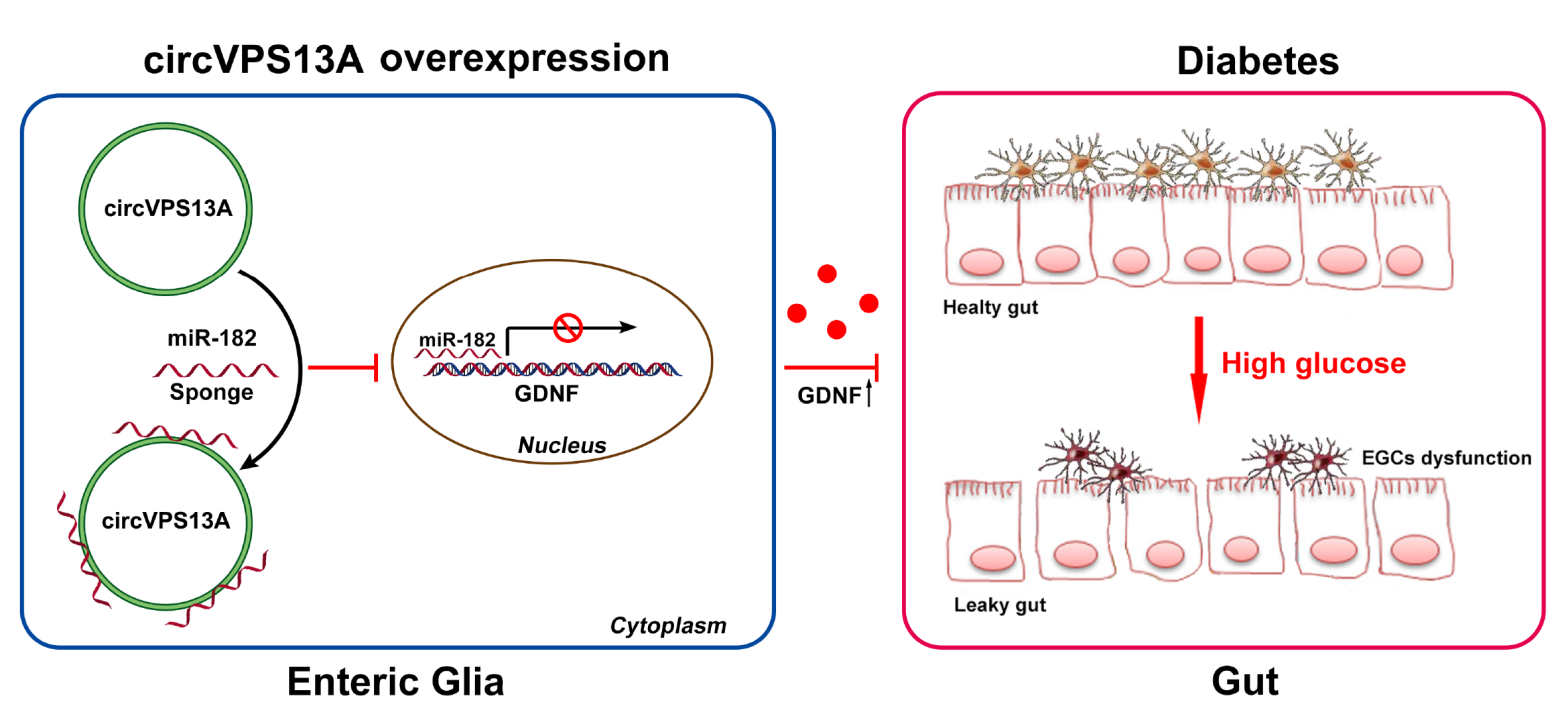
Figure 8 Diagram of the proposed mechanism of the effect of circVPS13A overexpression on diabetes-induced EGC damage Overexpression of circVPS13A increases cell viability and inhibits apoptosis of EGC by sponging miR-182 and inducing GDNF release.

In conclusion, in this study we revealed that circVPS13A is an important regulator of EGC damage in DM. circVPS13A overexpression increases cell viability and inhibits apoptosis of EGC by sponging miR-182 and inducing GDNF release ( Figure 8). Therefore, targeting the circVPS13A-miR-182-GDNF network may be a promising strategy for the treatment of DM-related GI complications.
